# Predicting recovery at home after Ambulatory Surgery

**DOI:** 10.1186/1472-6963-11-269

**Published:** 2011-10-13

**Authors:** Juan Viñoles, Maía V Ibáñez, Guillermo Ayala

**Affiliations:** 1 Ambulatory Surgery Unit. Hospital Universitario Dr. Peset, Avda Gaspar Aguilar 90, 46017 Valencia, Spain; 2 Department of Mathematics. Universitat Jaume I, 12071 Castellón, Spain; 3 Department of Statistics and Operational Research. Universidad de Valencia. Avda Vicent Andrés Estellés, 1, 46100 Burjassot, Spain

## Abstract

The correct implementation of Ambulatory Surgery must be accompanied by an accurate monitoring of the patient post-discharge state. We fit different statistical models to predict the first hours postoperative status of a discharged patient. We will also be able to predict, for any discharged patient, the probability of needing a closer follow-up, or of having a normal progress at home.

**Background:**

The status of a discharged patient is predicted during the first 48 hours after discharge by using variables routinely used in Ambulatory Surgery. The models fitted will provide the physician with an insight into the post-discharge progress. These models will provide valuable information to assist in educating the patient and their carers about what to expect after discharge as well as to improve their overall level of satisfaction.

**Methods:**

A total of 922 patients from the Ambulatory Surgery Unit of the Dr. Peset University Hospital (Valencia, Spain) were selected for this study. Their post-discharge status was evaluated through a phone questionnaire. We pretend to predict four variables which were self-reported via phone interviews with the discharged patient: sleep, pain, oral tolerance of fluid/food and bleeding status. A fifth variable called phone score will be built as the sum of these four ordinal variables. The number of phone interviews varies between patients, depending on the evolution. The proportional odds model was used. The predictors were age, sex, ASA status, surgical time, discharge time, type of anaesthesia, surgical specialty and ambulatory surgical incapacity (ASI). This last variable reflects, before the operation, the state of incapacity and severity of symptoms in the discharged patient.

**Results:**

Age, ambulatory surgical incapacity and the surgical specialty are significant to explain the level of pain at the first call. For the first two phone calls, ambulatory surgical incapacity is significant as a predictor for all responses except for sleep at the first call.

**Conclusions:**

The variable ambulatory surgical incapacity proved to be a good predictor of the patient's status at home. These predictions could be used to assist in educating patients and their carers about what to expect after discharge, as well as to improve their overall level of satisfaction.

## Background

Ambulatory Surgery (AS) is a routine clinical practice. In recent years, more complex operations have been included in ambulatory practice, and this development must be accompanied by a better monitoring of the patient's post-discharge status. Discharging an AS patient does not meant he/she has been cured. At that moment, instead of remaining in hospital, he/she starts the recovery phase at home. So it is very important to be able to predict his/her progress during the first hours following discharge. This information will help the physician to decide whether or not to discharge the patient, and will help the patients and their carers to know what to expect after discharge.

The majority of the studies found in the literature analyze the most frequent post-operative symptoms during the first 24 hours after AS, and signal the persistence of severe post-operative pain in these patients as the main factor to control [1,2]. Organisms like the "Joint Commission on Accreditation of Health Care Organizations" (JCAHCO) establish two ways to follow up the post-discharge status of a patient: extra-hospital assistance by inpatient units at home (or by a general practitioner or nurse) and protocol phone calls from the same Ambulatory Surgery Unit (ASU) [3]. Due to its convenience, immediacy and reduced cost, the phone call is the most commonly used alternative [1,4,5]. Several studies show that regular telephone calls improve overall user satisfaction being an effective tool to complete post-operative information, which is often unknown or not properly understood by the patient at the time of discharge. It allows the patient to receive advice about hygiene, health and diet and guidelines to decrease the consumption of analgesics [6]. It also becomes necessary to detect the need for read-mission into hospital for making a closer follow up of his/her evolution at home. The literature and our daily AS clinical practice seem to indicate that the status of the discharged patient can be well assessed by considering pain, oral tolerance, nausea, bleeding and other factors such as his/her psychological status, constipation, urine retention, sore throat or headache, onset of fever or how he/she complies with the treatment [7]. The protocol information for all these issues and their repeated evaluation during the days following discharge can help to control the post-operative status of the patient at home in conditions similar to hospitalization. With this purpose in mind, we have designed a standardized phone questionnaire, which is presented in Section 2.

Our main objective in this paper, is to predict the evolution of an AS patient during the first 48 hours following discharge, from the information provided by a set of variables known beforehand and another set of variables observed during the surgical procedure. In our hospital, the status of a discharged patient is controlled from the observation of four variables which are self-reported via phone interviews with the discharged patient. These ordinal variables are: sleep, pain, oral tolerance of fluid/food and bleeding status. Additionally, an aggregated value of these variables called the phone score (PS) is also defined. PS will become a global summary of the patient's status of the patient. All these variables, in turn, can be predicted using other predictors that are available prior to ambulatory surgery and during the ambulatory surgical process. So the statistical model will provide the physician with an insight into the progress of each patient at home. Different papers found in the literature consider age, sex, ASA, type of surgical intervention, surgical time, time from discharge and type of anaesthesia as the variables with most influence on post-discharge symptoms [2,5,8,9]. In our study, we have used all these variables but in order to get a standardization of data, and looking for a better predictive power, we have changed the variable "type of surgical intervention" with the variables: surgical specialty and ambulatory surgical incapacity (ASI), which will be defined in Section 2.

## Methods

Our data set consists of the observations taken on 922 patients who underwent operations in the ASU of the Dr. Peset University Hospital (Valencia, Spain), from October 8th 2003 to July 31st 2004. The selection criteria were patients from Gynaecology, General Surgery, ENT, Orthopaedic Surgery, Urology and Stomatology. All patients were selected for routine elective surgery, having met pre-operative selection criteria (American Society of Anesthesiologists criteria). They were judged to be fit enough to undergo surgery and to return home on the same day, having passed the post-anaesthetic discharge scoring system (PADSS) criteria [1]. All of them were treated with general or regional anaesthesia (peripheral blocks) or local monitored anaesthesia care (MAC). This study was approved by the Ethical Committee for Clinical Trials at the Dr. Peset University Hospital.

The data available on each patient were obtained from three different sources: (i) Variables known before admission of the patient into hospital, which will be called **pre-AS variables**; (ii) Variables observed during the operation and before discharge (intra-hospital variables), which will be called **AS variables**; and (iii) Variables describing the status of the patient after discharge, which will be called **post-AS variables**. This last set of variables was collected by a phone questionnaire.

As pre-AS variables, we considered: (i) **Age **(in years); (ii) **Gender**; (iii) **ASA status **(ASA), which is the anaesthetic risk according to the American Society of Anaesthetists [10]. Although that Society establishes five levels of risk, only patients in the first three levels are considered for AS. So "ASA status" will be a categorical variable with three levels; (iv) **Surgical specialty**: Gynaecology, General and Digestive Surgery, ENT, Orthopaedic Surgery, Stomatology and Urology; and (v) **Type of anaesthesia**: local Monitored Anaesthesia Care (MAC), regional anaesthesia (peripheral blocks, not spinal in this study) and general anaesthesia.

The second block of variables includes those observed during the patient's stay in hospital, and will be called AS variables. These variables are **surgical time**, defined as the duration of the operation, **discharge time **or the time between the end of the operation and discharge, including in the Post Anaesthesia Care Unit (PACU) I and PACU II recovery times, and **ambulatory surgical incapacity (ASI)**. This new variable, ASI, is an ordinal variable which tries to anticipate the level of incapacity at home and the severity of post-AS symptoms as a consequence of the surgical procedure. The definition of this new variable is motivated by the analgesia needs associated with a given surgical procedure, jointly with the expected inabilities of the discharged patient in his/her daily activity. It is really important to remark the anticipated character of this variable. The variable takes three different ordinal levels. Let us give a short explanation of each ASI level.

**High incapacity **(ASI = 3). Difficulty in mobility due to the existence of moderate pain. Impossibility of performing the basic tasks of hygiene and nutrition without aid for three or four days after discharge. Patient needs a combination of opioid, non-steroidal anti-inflammatory drugs, paracetamol and or infiltration with long term local anaesthesics.

**Medium incapacity **(ASI = 2). The patient is not able to carry out all the usual daily personal tasks at home. There is a medium level of pain and the need for a combination of non-steroidal anti-inflammatory drugs and paracetamol or similar.

**Low incapacity **(ASI = 1). The patient is able to carry out the basic tasks of daily cleanliness, hygiene and nutrition without aid. A care assistant is not necessary after discharge home. Either no analgesia is required or paracetamol.

As stated above, the definition of this variable is based on the kind of analgesia recommended by WHO in [11,12] adapted to ambulatory surgery, jointly with the expected inabilities of the discharged patient in his/her daily activity.

The surgical procedures scheduled in this study are detailed in Table 1, grouped by surgical specialty and ASI. The main difference between our variable ASI and the variable "type of intervention", is that we are aware that the same operation, depending among other things on the technique used (and on the general health status of the patient), usually corresponds to different levels of symptoms at home and therefore should be associated with a different postoperative patient evolution pattern: for instance, in our opinion, the surgical treatment of a varicose vein with stripping and spinal anaesthesia corresponds to an ASI level different from haemodynamic correction with local MAC, but the type of intervention is the same: surgical treatment of varicose veins.

**Table 1:** Surgical interventions grouped by surgical specialty and ASI

GYNAECOLOGY
**Low incapacity**

· Echographically guided puncture of ovarian cyst under sedation
· Excision of lesions in the vulva or vagina under sedation
· Diagnostic hysteroscopy under sedation

**Middle incapacity**

· Cervical conization.
· Surgical resection by hysteroscopy.
· Treatment of injuries to the vulva or vagina under general anaesthesia.
· Hydrolaparoscopic tubal exploration.

**High incapacity**

· Laparoscopic tubal and ovarian surgery.
· Laparoscopic management of endometriosis.
· Laparoscopic adnexectomy.
· Laparoscopic myomectomy.
· Laparoscopic lysis of adhesions.
· Laparoscopic sterilization.

**GENERAL AND DIGESTIVE SURGERY**

**Low incapacity**

· Excision benign pathology of breast under sedation.
· Excision of great lipomas under sedation.
· Excision of non-benign breast pathology under sedation.

**Middle incapacity**

· Pilonidal sinus excision under sedation.
· Umbilical hernia repair.
· Perianal pathology excision under sedation.

**High incapacity**

· Hemorroidectomy.
· Pre-peritoneal laparoscopic hernia repair.
· Open hernia repair.

**EAR, NOSE AND THROAT**

**Low incapacity**

· Excision of benign ENT pathology under sedation.
· Endolaryngeal microsurgery.
· Adenoidectomy and ear revision in adults and children.
· Videofibrosomnoscopy.

**Middle incapacity**



**High incapacity**

· Septoplasty
· Uvulopalatoplasty

**STOMATOLOGY**

**Low incapacity**



**Middle incapacity**

· Stomatologic treatment in psychic disabled under general anaesthesia.
· Stomatologic treatment under general anaesthesia.
· Stomatologic treatment under sedation.

**High incapacity**



**ORTHOPAEDICS**

**Low incapacity**

· Excision of injuries or benign tumours in limbs.

**Middle incapacity**

· Extraction of osteosynthetic material in arms or feet.
· Surgical interventions in tendon sheath, ganglion cyst, carpal and tarsal tunnel.
· Arthroscopy of knee (meniscopathy and cleaning in arthrosis).

**High incapacity**

· Acromioplasty.
· Arthroscopy of knee with ligamentoplasty.
· Osteotomy in hands or arms.
· Osteotomy in feet (hallus valgus, hallus rigidus, etc).

**UROLOGY**

**Low incapacity**

· Circumcision under sedation or general anaesthesia in adults or children.
· Change or withdrawal of urethral catheters.
· Cystoscopy.
· Prostate biopsy under sedation.
· Extracorporeal shock wave lithotripsy under sedation.

**Middle incapacity**

· Surgical interventions involving testicles (hydrocelectomy, varicocelectomy).
· Undescendent testicle.

**High incapacity**

Note that the different values of the variable ASI take into account the level of need for analgesics. Obviously, the definition of these levels uses our experience but is based on common practice given in the specialized literature. Different countries use different analgesics. Each group of analgesics has an application for each pain level. In our opinion, subjectivity in ASI definition is maintained at a minimum because it is based on pain patterns. This is the corner stone of our approach.

The last subset of variables consists of those needed to describe the status of a discharged patient, which we call Post-AS variables. In our hospital, AS is performed in the mornings and patients are discharged before 3 pm. The patient is called (by the same nurse) at 7 pm on the same day. The second and third phone calls are performed at the same time on the second and third days if necessary. The same trained nurse makes all the calls and reports the results of the questionnaire to the anaesthetist who must decide on the need for additional measures.

According to the literature, the types of procedures performed and the conclusions extracted from our own experience in AS, we consider a total of four outcomes, that will be called Post-AS variables: sleep, pain, oral tolerance and degree of bleeding, which have been categorized into four levels each:

**Sleep**: Very good status, normal sleep (2); good status but worried, not good sleep (1); anxious, bad sleep, drowsiness (0); and bad general status, dyspnoea, coma and paleness (-1).

**Pain**: Absence of pain, VAS 0-1, (2); moderate-mild pain, VAS 2-4 (1); severe pain, VAS 5-7, (0); and not accepted pain, VAS ≥ 8, (-1).

**Oral Tolerance**: Complete (2); only liquids (1); nausea, dizziness (0); and continuous vomiting (-1).

**Bleeding**: Absence (2); normal bleeding (1); abnormal bleeding but not abundant (0); and abundant bleeding (-1).

If any Post-AS variable takes the value -1, then the patient requires medical assistance. This value is included here in order to consider all possibilities. No such value has been observed for any patient in our study i.e. the real values for any variable goes from 0 to 2.

It is very important to note that these variables are self-reported. We are aware that we work with subjective responses. Some comments should be made: (i) We are using a procedure from Telemedicine where all patients can be monitored at home. No health personnel has to attend at home, with the corresponding saving of time. (ii) The only person who perfoms this phone questionnaire is an experienced nurse. She tries to reduce subjectivity by means of additional questions. (iii) She is supervised (by phone) by an anaesthetist specializing in ambulatory surgery who knows the majority of the patients called.

We have considered the sum of these four ordinal variables: the Phone Score (PS), as a simple aggregation measure. This variable has been devised as a global measurement of the patient's Post-AS recovery at home and tries to reflect the evolution of the patient at home on an ordinal scale. The definition of the four Post-AS variables enables us to say that a PS ranging between 4 and 8 indicates normal progress. A PS ranging between 0 and 3 indicates the need for closer follow-up. If any Post-AS variable is negative then the patient requires medical assistance and PS is not used.

Although the main emphasis in the paper concerns the four Post-AS variables, we thought that it would be reasonable to use an aggregated measure. The simplest choice would be to aggregate by giving the same weight to each one. The ordinal value is used as a number and the final aggregated value is the sum of the four original variables (considered as numbers). Note that when the ordinal values are used as numbers, we assume the same separation between the four consecutive categorical levels.

When the value obtained for PS at the first phone call signals normal progress, a second call is performed 24 hours later to assess this normal status. When a value ranging between 0 and 3 is obtained for PS at the first call, a second call is also performed 24 hours later to monitor the evolution of the patient. A third and fourth phone call can also be made with a lapse of 24 hours if the patient continues presenting problems, if he/she needs advice (guidelines) from the physician or if there is need for psychological help for patients and/or caretakers.

As stated above, our main aim in this paper is to build a predictive model for the Post-AS variables as a function of the pre-AS and AS variables. In order to achieve this, a generalized linear model (in particular, a proportional odds model explained in Section 2.1) will be fitted for each response. After fitting the model, it will be possible to predict the patient's Post-AS status from several variables routinely used in Ambulatory Surgery.

### Statistical analysis

As stated above, our aim is to build a predictive model of five different multinomial ordinal variables (sleep, pain, oral tolerance, degree of bleeding and phone score) of AS patients during the first 48 hours following discharge, as a function of another eight explicative variables. Three of these explicative variables are numerical (age, surgical time and discharge time), and the other five are considered as categorical: gender (with 2 levels), ASA status (with 3 levels), surgical specialty (6 levels), ambulatory surgical incapacity (3 levels) and type of anaesthesia (3 levels).

The most usual statistical model for this kind of data is the ordinal regression model, in particular the proportional odds model [13,14] Let us briefly recall this well-known model. Let *Y *be an ordinal response with *J *possible categories, *Y *∈ {1, 2, · · ·, J}. Let *x *= (*x*_1_, *x*_2_, · · ·, *x*_*d*_) be the set of predictive (independent) variables. Then the proportional odds model is defined from the following conditional distribution functions as:

(1) $$\begin{array}{l} logit(P(Y\leq j|x))=log\frac{P(Y\leq j|x)}{P(Y>j|x}=\\ =\alpha_{j}+\sum_{i=1}^{d}\beta_{i}x_{i},\;\;j=1,2,\cdots ,J-1 \end{array}$$

The parameters α_*j*_'s and *β*_*i*_'s are estimated by maximizing the likelihood function given by

(2) $$\begin{array}{c} L\left(\alpha_{1},\ldots ,\alpha_{J-1},\beta_{1},\ldots ,\beta_{d}\right)=\\ \;\;\overset{n}{\underset{i=1}{\prod }}\left[\overset{J}{\underset{j=1}{\prod }}\left(\frac{\exp\left(\alpha_{j}+\sum_{i=1}^{d}\beta_{i}x_{i}\right)}{1+\exp\left(\alpha_{j}+\sum_{i=1}^{d}\beta_{i}x_{i}\right)}-\right)\right)\\ \left({\left(\;\;\;\;\frac{\exp\left(\alpha_{j-1}+\sum_{i=1}^{d}\beta_{i}x_{i}\right)}{1+\exp\left(\alpha_{j-1}+\sum_{i=1}^{d}\beta_{i}x_{i}\right)}\right)}^{z_{ij}}\right], \end{array}$$

where *z*_*ij *_= 1 if *y*_*i *_= *j *and zero otherwise. A detailed presentation of this model can be found in Chapter 7 of [13].

A stepwise procedure, based on the Akaike Information Criterium (AIC) is used to select the best subset of variables to be considered in each model. Let us remember that the Akaike Information Criterium (AIC) is defined as: **AIC **= **-2 **(**maximized log likelihood - number of parameters in model**).

**Figure 1:**
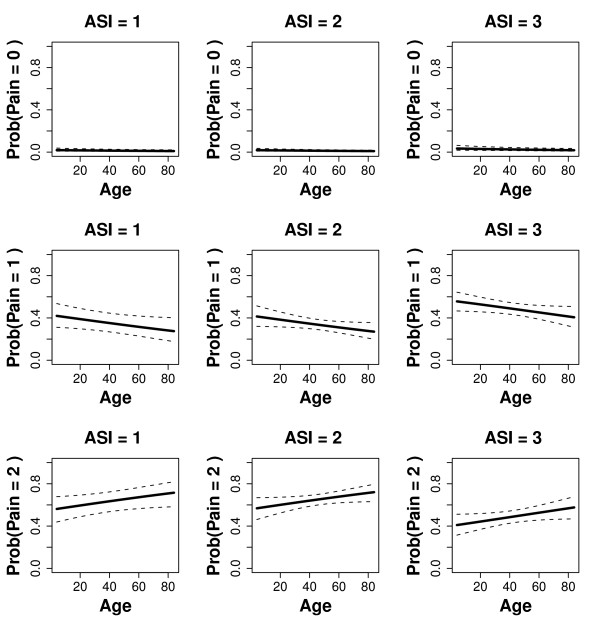
**Display of the interaction between age and ambulatory surgery incapacity (ASI), showing point-wise 95% confidence intervals around the fitted probabilities**.

In our case, the number of parameters in the model is equal to (*J *- 1) + *d*. Once the parameters in Eq.1 have been estimated, we will be able to predict *P*(*Y *≤ *j*|*x*), i.e. the probability of a patient being in any of the possible Post-AS categories up to and including the cut point category j. Really, from a clinical point of view, we would use the probability of a given value *j *i.e. *P*(*Y *= *j*|*x*) = *P*(*Y *≤ *j*|*x*) - *P*(*Y *≤ *j *- 1|*x*) for *j *= 2,..., *J *and *P*(*Y *= 1|*x*) = *P*(*Y *≤ 1|*x*). The anaesthetist could base his/her discharge decision on the probabilities predicted by the models fitted (see Tables 2, 3, 4, 5 and 6). For instance, Figure 1 displays the fitted probabilities by taking into account age (abscisas) and the different ASI values.

**Table 2:** Sleep at first, second and third call

First call			
**Covariates**	**Coefficients**	**Fitted values**	**Standard Error**

	α_**1**_	-4.1840	0.3506
	α_**2**_	0.2816	0.1586
Age	*β*_*Age*_	-4.1840	0.0044
Sex	*β*_*Sex*_	0.2816	0.1586

AIC	1093.197		

Cross-Validation	0.4069		


Second call			

Covariates	Coefficients	Fitted values	Standard Error

	α_**1**_	-4.0452	0.3631
	α_2_	-0.3072	0.2825
Surgical Specialty	*β_General Surgery_*	-0.5127	0.2304
	*β*_*ORL*_	-0.8455	0.3130
	*β*_*Stomatology*_	0.5728	0.3653
	*β*_*OrthopedicSurgery*_	0.2319	0.2631
	*β*_*Urology*_	-0.9386	0.5083
Age	*β*_*Age*_	-0.0030	0.0053
ASI	*β*_*ASI.Middle*_	-1.0422	0.2254
	*β*_*ASI.High*_	0.0117	0.1887

AIC	2112.351		
Cross-Validation	0.4327		


Third call			

Covariates	Coefficients	Fitted values	Standard Error

	*β*_**1**_	-1.8670	0.6104
	*β*_**2**_	1.3105	0.5735
Age	*β*_*Age*_	0.0215	0.0126
Anaesthesia	*β*_*Peripheralblock*_	0.0017	0.7118
	*β*_*MAC*_	14.6685	1.3e-07

AIC	209.000		
Cross-Validation	0.3983		

Coefficients and standard errors of fitted models. AIC and CV.

**Table 3:** Pain at first, second and third call

First call			
**Covariates**	**Coefficients**	**Fitted values**	**Standard Error**

	α_0_	-3.7291	0.3374
	α_1_	-0.0179	0.2066
Age	*β*_*Age*_	0.0084	0.0045
ASI	*β*_*ASI.Middle*_	-0.4345	0.1698
	*β*_*ASI.High*_	-0.2713	0.1356

AIC	1084.006		
Cross Validation	0.4635		


Second call			

Covariates	Coefficients	Fitted values	Standard Error

	α_0_	-2.8014	0.3943
	α_1_	0.7450	0.3519
Surgical Specialty	*β_General Surgery_*	-0.6266	0.2728
	*β*_*ORL*_	0.0437	0.3195
	*β*_*Stomatology*_	1.2886	0.4325
	*β*_*OrthopedicSurgery*_	0.4119	0.3350
	*β*_*Urology*_	0.0837	0.5450
Age	*β*_*Age*_	0.0178	0.0055
Sex	*β*_*Sex*_	0.0215	0.2097
Anaesthesia	*β_Peripheralblock_*	-0.2592	0.3241
	*β*_*MAC*_	-1.1872	0.5100
ASI	*β*_*ASI.Middle*_	-0.7485	0.2280
	*β*_*ASI.High*_	-0.4945	0.1933

AIC	966.627		
Cross Validation	0.4161		


Third call			

Covariates	Coefficients	Fitted values	Standard Error

	α_0_	-1.9296	0.5959
	α_1_	1.1947	0.5497
Age	*β*_*Age*_	0.0142	0.0125

AIC	211.565		
Cross Validation	0.4273		

Coefficients and standard errors of fitted models. AIC and CV.

**Table 4:** Tolerance at first, second and third call

First call			
**Covariates**	**Coefficients**	**Fitted values**	**Standard Error**

	α_1_	-3.3433	0.3952
	α_2_	0.2667	0.3669
Surgical Specialty	*β*_*General Surgery*_	0.6192	0.2765
	*β*_*ORL*_	2.9448	0.3599
	*β*_*Stomatology*_	0.3393	0.3615
	*β*_*OrthopedicSurgery*_	2.6205	0.3822
	*β*_*Urology*_	1.8661	0.4465
Age	*β*_*Age*_	-0.0151	0.0054
Sex	*β*_*Sex*_	-0.1508	0.2214
Anaesthesia	*β*_*Peripheralblock*_	0.9934	0.3560
	*β*_*MAC*_	-0.6094	0.5667
ASI	*β*_*ASI.Middle*_	-0.9810	0.2502
	*β*_*ASI.High*_	0.1343	0.1933

AIC	1006.784		
Cross Validation	0.3057		


Second call			

Covariates	Coefficients	Fitted values	Standard Error

	*α*_1_	-3.4939	0.6040
	*α*_2_	-1.3215	0.5107
Surgical Specialty	*β*_*General Surgery*_	1.3791	0.4522
	*β*_*ORL*_	1.3573	0.4903
	*β_Stomatology_*	-0.6681	0.6349
	*β*_*OrthopedicSurgery*_	1.5133	0.6329
	*β*_*Urology*_	12.6685	9.9e-08
Age	*β*_*Age*_	0.0113	0.0011
ASI	*β*_*ASI.Middle*_	-0.3343	0.3924
	*β*_*ASI.High*_	-1.2381	0.3946

AIC	411.505		
Cross Validation	0.1334		


Third call			

Covariates	Coefficients	Fitted values	Standard Error

	*α*_1_	-3.4939	0.6040
	*α*_2_	-1.3215	0.5107
Surgical Specialty	*β*_*General Surgery*_	19.9142	0.4522
	*β*_*ORL*_	19.8182	0.4903
	*β*_*Stomatology*_	0.6934	0.6349
	*β*_*OrthopedicSurgery*_	20.0539	0.6329
	*β*_*Urology*_	20.3581	9.9e-08
Sex	*β*_*Sex*_	20.7398	0.0011

AIC	63.69		
Cross Validation	0.1012		

Coefficients and standard errors of fitted models. AIC and Cross-Validation.

**Table 5:** Degree of bleeding at first, second and third call

First call			
**Covariates**	**Coefficients**	**Fitted values**	**Standard Error**

	*α*_1_	-6.0473	0.5117
	*α*_2_	-1.2185	0.3915
Surgical Specialty	*β*_*General Surgery*_	3.4687	0.4134
	*β*_*ORL*_	-1.6315	0.3848
	*β*_*Stomatology*_	2.1440	0.3918
	*β*_*orthopedicSurgery*_	17.9220	4.3e-08
	*β*_*Urology*_	3.4628	0.4830
Age	*β*_*Age*_	-0.0165	0.0007
ASI	*β*_*ASI.Middle*_	-2.3919	0.3520
	*β*_*ASI.High*_	3.1268	0.3138

AIC	642.15		
Cross Validation	0.18044		


Second call			

Covariates	Coefficients	Fitted values	Standard Error

	*α*_1_	-5.9866	0.6970
	*α*_2_	-0.7807	0.3812
Surgical Specialty	*β*_*General.Surgery*_	2.2251	0.3394
	*β*_*ORL*_	-1.0804	0.3545
	*β*_*Stomatology*_	2.7194	0.4928
	*β*_*OrthopedicSurgery*_	4.0566	0.7328
	*β*_*Urology*_	2.3690	0.7971
Age	*β*_*Age*_	0.0003	0.0074
ASI	*β*_*ASI.Middle*_	-1.6307	0.3338
	*β*_*ASI.High*_	2.0055	0.2807

AIC	617.976		
Cross Validation	0.2054		


Third call			

Covariates	Coefficients	Fitted values	Standard Error

	*α*_2_	5.41e-02	2.33e-01
Surgical Specialty	*β*_*General Surgery*_	1.3322	5.51e-01
	*β*_*ORL*_	0.3514	6.86e-01
	*β*_*Stomatology*_	1.85e+01	2.06e+03
	*β*_*OrthopedicSurgery*_	1.85e+01	1.74e+03
	*β*_*Urology*_	1.85e+01	3.26e+03

AIC	153.01		
Cross Validation	0.1722		

Coefficients and standard errors of fitted models. AIC and Cross-Validation.

**Table 6:** Phone score at first, second and third call

First call			
**Covariates**	**Coefficients**	**Fitted values**	**Standard Error**

	*α*_**3**_	-4.1014	0.4085
	*α*_**4**_	-1.8458	0.2972
	*α*_5_	-0.2971	0.2871
	*α*_**6**_	1.2692	0.2924
	*α*_7_	2.8708	0.3078
Surgical Specialty	*β_General Surgery_*	1.2807	0.2728
	*β*_*ORL*_	1.1829	0.3195
	*β*_*Stomatology*_	1.2718	0.4325
	*β*_*OrthopedicSurgery*_	2.2590	0.3502
	*β*_*Urology*_	1.7431	0.5450
Age	*β*_*Age*_	-0.0051	0.0055
Sex	*β*_*Sex*_	-0.0220	0.2097
ASI	*β*_*ASI.Middle*_	-0.9810	0.2280
	*β*_*ASI.Hihg*_	0.1343	0.1933

AIC	2140.351		
Cross Validation	1.2569		


Second call			


Covariates	Coefficients	Fitted values	Standard Error

	*α*_**3**_	-4.7086	0.5071
	*α*_4_	-2.9425	0.3481
	*α*_**5**_	-1.1755	0.3137
	*α*_**6**_	0.3783	0.3104
	*α*_**7**_	1.7007	0.3174
Surgical Specialty	*β_General Surgery_*	0.3872	0.2212
	*β*_*ORL*_	-0.3763	0.2845
	*β*_*Stomatology*_	1.5222	0.3445
	*β*_*OrthopedicSurgery*_	1.1875	0.2499
	*β*_*Urology*_	0.4556	0.4830
ASI	*β*_*ASI.Middle*_	-1.2186	0.2075
	*β*_*ASI.High*_	0.1299	0.1712

AIC	1825.098		
Cross Validation	1.5346		


Third call			

Covariates	Coefficients	Fitted values	Standard Error

	*α*_**3**_	-4.3577	0.8924
	*α*_4_	-3.4107	0.7056
	*α*_**5**_	-1.1511	0.5748
	*α*._**6**_	0.3421	0.5764
	*α*_**7**_	1.7897	0.5975
Age	*β*_*Age*_	0.0190	0.0112
ASI	*β*_*ASI.Middle*_	-2.2458	0.9085
	*β*_*ASI.High*_	1.4220	0.5657

AIC	356.0507		
Cross Validation	1.2797		

Coefficients and standard errors of fitted models. AIC and Cross-Validation.

We will evaluate which set of variables taken together are more relevant to predict the subsequent patient status. These variables should be considered jointly in order to provide information concerning the advisability of medical discharge. This could be a useful tool in clinical practice.

Two different strategies have been used to evaluate the goodness-of-the-fit. First, although the AIC criterion could be used, we have preferred to work with *Cross-Validation *(CV) which is a forecast-based criterion defined as:

$$CV=\frac{1}{n}\overset{n}{\underset{i=1}{\sum }}{\left(y_{i}-{ŷ}_{-1}\right)}^{2}$$

where *y*_*i *_denotes the *i *- *th *observation of the response variable and ${ŷ}_{-i}$ denotes the value predicted by the model, fitted by using all the observed values except the *i *- *th *observation.

A second procedure to check the proportional-odds assumption is the score test. It has been used an approximation of this score method that can be found in [15,16].

All the statistical analyses will be performed by using R [17], a free software application for statistical computation and graphics. One of the packages in R, called MASS [15], contains many of the functions used in this paper.

## Results

A proportional odds model (Eq. 1) is fitted for each outcome (sleep, pain, oral tolerance, degree of bleeding and PS) and phone call. As can be seen in Table 7, the number of patients who have needed a fourth, fifth and/or sixth call is really very small (26 patients, 1.4%), so only the data obtained in the first three phone calls will be used in this analysis. There are therefore 15 models to fit.

**Table 7:** Joint distribution of the number of phone calls per patient and the scores obtained

Score	3	4	5	6	7	8	Total
First call	13	89	199	276	219	126	922
Second call	7	33	133	234	192	162	761
Third call	2	3	34	45	34	20	138
Fourth call	0	0	4	6	8	2	20
Fifth call	0	1	0	1	2	0	4
Sixth call	0	0	2	0	0	0	2

	22	126	372	562	455	310	1847

A stepwise procedure based on the AIC criteria has been used to select the subset of covariates which are significant for each fit. The coefficients of all the remaining variables for each model can be seen in Tables 2, 3, 4, 5 and 6. At the first call, the non-significance of variables whose importance has traditionally been taken for granted, such as ASA status, Surgical Time or Discharge Time, should be emphasized. In our models, they are dwarfed by the presence of the variables stated above, ASI and surgical specialty. At the second call, ASA status, Surgical Time or Discharge Time are once again dwarfed in the models. The data from the third call represents 7.4% of all data. The time elapsed since the operation makes the response variables less sensitive to the predictors. Moreover, the third call has a small sample size and the tests are not very powerful. It can be said that the covariates ASA, Surgical Time and Discharge Time do not appear as significant in any model. In our experience, Surgical Time and Total Post-Surgical Time describe the operation and possible indirect complications which are not observed directly in the first few hours after discharge. On the other hand, ASI is significant for all responses at the first and second call, except for the variable sleep at the first call. All fitted models are explained and interpreted in a detailed way in [7]. To illustrate this, let us analyze the results obtained for pain at the first phone calls.

Among all the post-operative symptoms, pain is one of the most widely analyzed in the literature. Moderate-severe pain and the indirect effect of analgesic treatments are the most common problems detected in the post-operative status of AS, as is shown in [2,5,8,9]. Uncontrolled pain is the main cause of discharge delays [10], multiple visits to the medical practitioner [6], hospitable visits to emergencies [18], and readmission to hospital [19]. Table 8 shows the percentage of patients in these studies with nausea and severe or moderate pain during the first hours after discharge, along with the percentages obtained in our data set. Similar rates are obtained from the five samples despite the diversity of the data and the fact that the studies span more than a decade. Therefore, it can be concluded that although our study is an observational one, we are getting analogous responses to those obtained in other clinical trials.

**Table 8:** Comparative (descriptive) analysis of the conclusions of several authors who have analyzed the Post-AS status of the patients

Main post-operative symptoms according with					
	**Chung'97**	**Rawal'97**	**McGrath'04**	**Mattila'05**	**Vinoles'10**

Patients	10008	1100	5703	2754	922
Severe pain	5.3%	10%	10%	18%	7.1%
Moderate-mild pain		25%	21%	27%	21.6%
Nausea		20%		23%	10.1%

Predictive factors that have a significant influence on the Post-AS symptoms in accordance with					

	Chung'97	Rawal'97	McGrath'04	Mattila'05	Vinoles'10

Age	Yes	Yes	Yes	Yes	Yes
Sex	Yes	Yes	Yes	Yes	Yes
ASA	Yes				No
Type of Anaesthesia		Yes		Yes	Yes
Surgical time	Yes		Yes	Yes	No
Type of surgery	Yes	Yes	Yes	Yes	Yes

Types of operations that cause major degree of PS pain in accordance with					

	Chung'97	Rawal'97	McGrath'04	Mattila'05	Vinoles'10

	Orthopaedics	Hernia repair	Microdiscectomy	Orhopaedics	High ASI:
	Urology	Orhopaedic	Laparoscopic	Long duration surgery	Laparoscopic
	General surgery	Hand surgery	Laparosc. cholecystectomy		Gynaecology
	Plastic surgery	Varicose vein surgery	Shoulder surgery		Hernia repair
			Elbow/hand surgery		Hemorroidectomy
			Ankle surgery		Septoplasty
			Inguinal hernia rep.		Orthopaedics

As has been said, "pain" is an ordinal variable with four categories. None of the patients in our data set had manifested an unbearable level of pain, so no information is available to predict that category. Therefore, the variable "pain" is considered in PS as a categorical variable with just three categories: Pain = 0 severe pain; Pain = 1 (moderate-mild); pain = 2 (absence of pain). As shown in Table 2, significant covariates for pain at the first call are age and ASI. This table shows the coefficients of these covariates in the fitted model along with the coefficients of the significant covariables for fitting pain at the second and third call. Non-significant covariates have been removed and do not appear in the table. Note that ASI is a categorical predictor with three categories, so its influence will be described by means of two dummy variables and therefore by three coefficients. By substituting all the coefficients in (Eq. 1), both *P*(*Pain *≤ 0|*data*) and *P *(*Pain *≤ 1|*date*) at each call can be obtained:

$$\begin{array}{c} P\left(Pain\leq 0|x\right)=\frac{exp0}{1+exp0},\\ P\left(Pain\leq 1|x\right)=\frac{exp1}{1+exp1},\\ P\left(Pain\leq 2|x\right)=1. \end{array}$$

with

(3) $$\begin{array}{l} exp0=\\ \;\;\;\;exp(-3.7291+0.0084\cdot age\\ \;\;\;\;\;-0.4345\cdot 1_{ASI.Middle}\\ \;\;\;\;\;\;\;\;\;\;-0.2713\cdot 1_{ASI.High}), \end{array}$$

(4) $$\begin{array}{l} exp1=\\ \;\;\;\;exp(-0.0179+0.0084\cdot age\\ \;\;\;\;\;-0.4345\cdot 1_{ASI.Middle}\\ \;\;\;\;\;\;\;\;\;-0.2713\cdot 1_{ASI.High}). \end{array}$$

From these equations, probabilities *P*(*Pain *= 0), *P*(*Pain *= 1) and *P*(*Pain *= 2) at the first phone call are easily calculated:

$$\begin{array}{c} P\left(Pain=0|x\right)=\;\;P\left(Y\leq 0|x\right),\\ P\left(Pain=1|x\right)=\;\;P\left(Y\leq 1|x\right)-P\left(Y=0|x\right),\\ P\left(Pain=2|x\right)=\;\;1-P\left(Y\leq 1|x\right). \end{array}$$

Odds ratio can now be computed, taking *Pain *= 0 as a reference value, but once again we will not obtain a single value but a function of the patient's age and ASI.

A graphical representation of the estimated probabilities *P*(*Pain *= 0), *P*(*Pain *= 1) and *P*(*Pain *= 2), as a function of the significant covariates, can be obtained by using effect displays (Figure 1). These plots represent a statistical model by showing carefully selected portions on its response surface. Effect displays were introduced in [20] for generalized linear models, and were implemented in R in [21]. Their extension to the proportional odds model from [22] is also implemented in R.

Figure 1 shows the plot of the fitted probabilities for each category of the response (pain at the first call). As ASI takes on only three values while age is continuous, a separate plot is built for each level of ASI and pain, placing age on the abscissas. The solid line in each plot represents the fitted probability of getting each response category and 95% point-wise confidence intervals are added as dotted lines around the fitted probabilities. Similar graphical representations can be plotted for variable pain at the second and third phone calls. They can be found in [7], along with a deeper analysis of each result.

As can be seen in Figure 1, the probability of having severe pain (pain = 0 in PS) is almost zero, independently of the level of ASI considered and of the patient's age. Operations with a higher ASI have a higher probability of getting higher values for variable pain. Between 27% and 42% of the patients with a low or a medium level of ASI reported having a mild-moderate level of pain (pain = 1 in PS) and for patients with a high level of ASI this percentage increases, ranging from 40% to 56%. Finally, the probability of patients with a low or medium level of ASI having an absence of pain (pain = 2 in PS) ranges from 56% to 72%, and for those with a high level of ASI this probability decreases to values from 41% to 58%. In addition to variable "pain", this analysis should be repeated for the remaining response variables. The complete analysis can be found in [7].

To evaluate the goodness-of-the-fit the AIC criterion could have been used, but instead we have preferred to work with the Cross-Validation (CV) because it is a forecast-based criterion. This index compares, one by one, each observation of the response variable with the value predicted by the model, fitted by using all the observed values excepting the one considered each time. The values obtained for each model are shown in Tables 2, 3, 4, 5 and 6, and guarantee the suitability of our models and the-goodness-of-fit.

An approximation of the score test has been used for all models. The p-values are not significant and so the assumption of proportional odds is reasonable for these data except for outcomes tolerance and bleeding at the first call. Anyway in order to maintain a coherence in the whole analysis and by considering the cross-validation results, we have used the same model for all fits.

## Discussion

The essence of ambulatory surgery is to have prior knowledge of the post-surgical evolution of each patient at home. It is not possible to discharge a patient if we do not know how he/she will evolve at home. Most of studies found in the literature that we have reviewed [2,5,8,9] focus the main post AS problems on the existence of postoperative pain and nausea. Our main objective in this paper has been to develop a statistical methodology for predicting the status of an AS patient during the first 48 hours after discharge from the information provided by variables routinely used in Ambulatory Surgery.

In our hospital, postoperative monitoring is carried out via a phone survey where four variables are self-reported by the discharged patient: sleep, pain, tolerance and bleeding. The results of this survey are added up and summarized into a single indicator of the evolution of the post-operative state, called phone score (PS). This new variable facilitates the existence of a clear and comprehensible indicator similar to those existing in other medical fields [23].

The decision to discharge a patient after surgery is based on the patient's condition at that time and on the anticipation of favorable evolution at home. The ASI quality criteria have been defined by several international organizations, like the Joint Commission on Accreditation of Healthcare Organizations [3], and the importance of ensuring a close follow-up of the progress made by discharged patients at home is recognized by all these international organizations.

Different studies found in the literature (Table 8) conclude that factors such as type of intervention, duration, age and sex strongly influence the post-operative symptoms after discharge. But these studies show differences between the surgical procedures that produce greater postoperative discomfort. It seems that most moderate - severe pain is attributed to orthopaedic surgery, followed by hernior-rhaphy, laparoscopy and other types of surgery lasting more than 59 minutes (Table 8). In our study, we have considered age, gender, ASA status, surgical specialty, type of anaesthesia, surgical time, discharge time and ASI, as possible predictors of the outcomes of interest. Thus, the type of surgical intervention has been characterized by means of two predicting variables: the surgical specialty and the ambulatory surgical incapacity. This second variable is defined in order to group the different AS interventions according to the expected post-operative status (expected degree of incapacity) of the patient. Our results show that this variable, along with the surgical specialty, will define the type of post-operative status for each type of patient with greater precision than the type of surgical procedure, surgical time or time of discharge. For example, if a hernia is going to be operated on different post-surgical needs and levels of incapacity. So, in our opinion, a variable "kind of intervention" including different surgical techniques cannot distinguish between those different post-operative needs of the patient in both the hospital and at home. Table 1 shows our purpose in grouping the different types of intervention into the three ASI levels.

Table 8 shows the percentage of patients with nausea and severe or moderate pain during the first hours after discharge in published studies that have been compared with our data set. Similar rates are obtained from the five samples despite the diversity of the data and the fact that the studies span more than a decade. Therefore, it can be concluded that although our study is an observational one, we are getting analogous responses to those obtained in other clinical trials, and that despite the resources available, the rates of postoperative pain have not decreased too much. Future studies should demonstrate whether the emergence of new drugs or less painful surgical procedures can improve these data.

Tables 2, 3, 4, 5 and 6, show the significant variables for predicting the different response variables in the three calls, and their coefficients in the different models. It must be noted that the ASA, operative time and discharge time are non significative in any of the responses (sleep, pain tolerance, bleeding and PS) at any time. The remaining covariates are significative to a greater or lesser degree as detailed in these tables. The lack of significance of ASA in the response variables is due to the observational character of our study, where the selection of patients prior to surgery leads to a bias toward every level of ASI with respect to ASA (among other variables). Patients with ASA III and high ASI are excluded from ambulatory surgery. That fact would explain this result.

Unlike other studies, we conclude that surgical time and discharge time are not significant in explaining the postoperative symptoms analyzed (Table 8). Significant differences were found between the times of different types of surgeries classified into the different ASI levels (*p *< 2.2 - 16). Interventions with longer surgical time are those labeled with higher ASI level. So, in an indirect way, the information gathered by the different times is collected by the new variable ASI, which together with the variable "specialty", have the greatest predictive power in our study.

A deeper explanation of the influence of different predictors on responses could be obtained by graphical representations. Unfortunately, the length of the article does not allow us to include too many plots, so we have included one of the most explicative.

Figure 1 shows the probability of occurrence of three levels of pain (no pain, mild - moderate and severe) depending on the age of the patient and his/her ASI level. It can be seen that the answer differs depending on the degree of ASI and that it is clearly modulated by age. A greater degree of ASI implies a greater probability of perceiving a higher level of pain. Age also increases the perception of pain and the need for care.

The predictive power of the variable "specialty" must also be noted. Tables 2, 3, 4, 5 and 6, show how it influences tolerance, bleeding and PS at the first call how it influences all responses at the second call and how it affects tolerance and bleeding at the third call. Several specialties such as gynaecology, dentistry and ENT, which usually imply bleeding, can influence the response in a natural way. It is normal that patients submitted to a hysteroscopy, septoplasty or removal of wisdom teeth can be bleeding for a few days. Regarding nausea, it is sometimes due to pain or side effects. At other times it caused by postoperative ileus after laparoscopic surgery and sometimes by swallowing blood after dental surgery or ENT. The influence of different predictor variables on the behavior of PS is shown in Table 6. In general, the likelihood of higher PS is associated with less aggressive surgery.

Thus, we have developed a statistical methodology with which to build the predictive model of these five variables (sleep, pain, oral tolerance, degree of bleeding and phone score) for AS patients during the first hours after discharge, as a function of the information at hand before discharge (pre-AS and AS covariates). The goodness-of-the-fit is guaranteed by the values obtained for the different cross-validations. Some of these predictive variables can be controlled (specialty, ASI, type of anesthesia) and others not (age, sex, ASA). Knowledge of these variables can help the physician to decide about whether to discharge the patient or not, and can also help the patients and their carers to know what to expect after discharge. Its main benefit is the possibility of having a tool to predict how a patient will evolve after discharge. It could be interesting to incorporate into the models described in this study as a friendly software tool.

## Conclusions

In conclusion, we can assess that it is possible to predict the postoperative status of a discharged patient in the first hours after discharge. Eight risk factors (pre-AS and AS predictors) have been considered, and the statistical model has detected the significant ones for four different outcomes and three different post-discharged times. Most of these predictors can be controlled before surgery and this will facilitate the decision about discharging a patient. We have found that the variables age, sex, anaesthesia type, surgical specialty and ASI are significant predictors for most of the outcomes considered, showing a great prediction power of the postoperative symptoms analyzed in this paper. The postoperative recovery of the patient is the main challenge and it justifies ambulatory surgery. If it is not monitored a patient's satisfaction can be diminished and so can confidence in the ambulatory surgery.

## Competing interests

The authors declare that they have no competing interests.

## Authors' contributions

JV collected the data and posed the original problem. The statistical analyses were performed by MVI and GA. The paper was written jointly. All authors read and approve the final manuscript.

## Pre-publication history

The pre-publication history for this paper can be accessed here:

http://www.biomedcentral.com/1472-6963/11/269/prepub
